# Adaptability in the Midst of Anatomical Challenges: A Case of Situs Inversus Totalis in Laparoscopic Cholecystectomy

**DOI:** 10.7759/cureus.58770

**Published:** 2024-04-22

**Authors:** Christopher Nguyen, David Schutter

**Affiliations:** 1 Medicine, Lincoln Memorial University, DeBusk College of Osteopathic Medicine, Knoxville, USA; 2 General Surgery, Premier Surgical, Knoxville, USA; 3 General Surgery, Tennova Healthcare, Knoxville, USA

**Keywords:** gallbladder disease, congenital abnormalities, symptomatic cholelithiasis, laparoscopic cholecystectomy, situs inversus totalis

## Abstract

Situs inversus totalis (SIT) is a rare and nonfatal congenital anomaly where there is a complete inversion of a patient's visceral organs. Throughout the patient's lifetime, they will encounter various challenges due to their unique anatomic variation. In this case, we report the treatment of symptomatic cholelithiasis in a 33-year-old female with comorbid SIT who underwent a laparoscopic cholecystectomy without postoperative complications. Despite the added layer of complexity in her presentation, we were able to perform the cholecystectomy laparoscopically with slight modifications to better accommodate her anatomical mirroring.

Modifications made in the form of bed positioning, trochar placement, and surgical team positioning prove that strategic operative planning is essential to optimizing outcomes for this unique patient population.

## Introduction

Situs inversus totalis (SIT) is an autosomal recessive condition involving the complete left-right reversal of a patient's visceral organs [1]. The incidence of SIT has been recorded to be 1:10,000 [2]. Though the condition is seemingly rare, its presentation comes with a unique twist to common abdominal pathologies that require additional investigation. In many cases, the abdominal pathologies will present with symptoms on the opposite side of “normal” due to the anatomical inversion [3]. When patients with SIT present for surgical management, a surgeon's adaptability is put to the test. Like most gallbladder diseases, the standard procedure to treat symptomatic cholelithiasis is a laparoscopic cholecystectomy [4]. The option of performing the procedure using an open approach is reserved for severely complex cases, patients with multiple comorbidities, the presence of severe infections, and when deemed necessary by the surgeon [5,6]. Discussions regarding the utilization of other modalities, including the use of robotics, a two-surgeon approach, and a "single-incision" approach, have emerged; however, none have proven to reign superior [7-9]. With everything opposite of the standard “usual,” the diagnosis and management of SIT patients are higher on the difficulty index versus patients without SIT [2]. When indicated, surgeons should proceed with the elective procedure and may choose to supplement their pre-surgical planning with the utilization of imaging studies such as CT, endoscopic retrograde cholangiopancreatography (ERCP), and magnetic resonance cholangiopancreatography (MRCP) [10]. Though vascular variations may exist, SIT patients typically only differ from normal patients in regard to anatomic mirroring [11].

In this case, we report the treatment of symptomatic cholelithiasis in a 33-year-old female with comorbid SIT who underwent a laparoscopic cholecystectomy without postoperative complications. The combination of meticulous preoperative planning, a review of imaging, and the positioning of the surgical team and equipment played a large role in our patient's successful postoperative recovery.

## Case presentation

**Figure 1:**
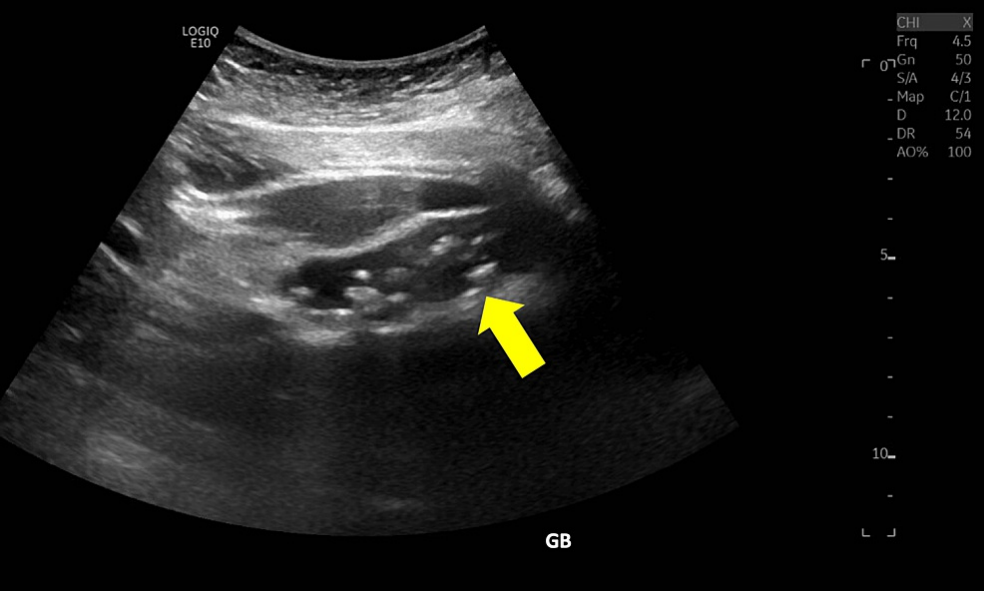
Ultrasound image of the left upper quadrant showing multiple calculi (yellow arrow) within the gallbladder

A 33-year-old female was referred to our clinic by her primary care physician and gastroenterologist with a recurrent history of episodic left upper quadrant pain and diffuse epigastric pain exacerbated by fatty foods. Over the past month, the patient states the episodes were occurring more frequently and felt they were beginning to impair her acts of daily living. A previous ultrasound demonstrated the presence of stones in her gallbladder; however, there was no mention of its localization to the left abdomen on the radiology report (Figure 1). Her past medical history was significant for SIT. She had no history of abdominal surgeries. Her preoperative labs were within normal limits. Due to her presentation, the patient was signed up for an elective laparoscopic cholecystectomy at the outpatient surgery center.

**Figure 2:**
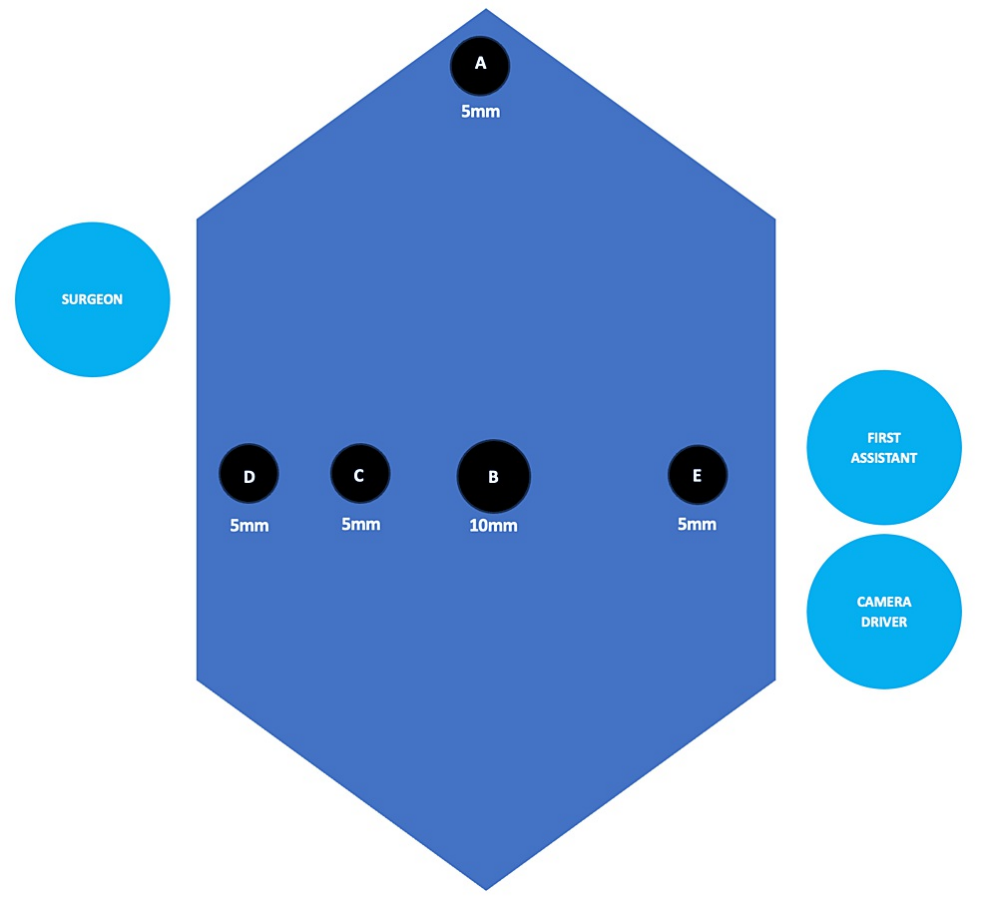
Trocar sites and operative team placement A: subxiphoid 5 mm, B: umbilical 10 mm, C: midclavicular line 5 mm, D: anterior axillary 5 mm, E: anterior axillary 5 mm Image Credit: Christopher Nguyen

On the day of surgery, the patient was taken back to the operating room and placed under general anesthesia. The abdomen was prepped and draped in the usual sterile fashion. A preoperative timeout was conducted, and all individuals in the room concurred. A small vertical incision was made superior to the umbilicus, and visual entry into the peritoneum was confirmed via laparoscope. A Hassan trocar was then utilized to insufflate the abdomen to a pressure of 15 mmHg. The laparoscope was then reinserted for abdominal examination and review of anatomical deviations. At this time, the patient was placed in the reverse Trendelenburg position with the right side tilted down. Under laparoscopic visualization, three standard 5-mm ports were placed: two in the right upper quadrant along the costal margins and one in the epigastrum. Upon further investigation of the biliary anatomy, the cystic duct appeared more medial as opposed to lateral. For ease of maneuvering, an additional 5-mm port was placed in the left flank region. In total, one Hassan trocar was utilized in addition to four additional 5-mm trocars (Figure 2). The gallbladder was then located on the left side of the patient's abdomen, grasped, and retracted superolaterally. The cystic duct and cystic artery were circumferentially dissected with no evidence of anatomic deviation in the biliary system. Once identified, the structures were clipped, twice on the proximal side and once distally, before being divided with scissors. Electrocautery was then used to divide the attachments between the gallbladder and the liver. During the dissection process, mild bleeding occurred. Electrocauterization was used to resolve this, in addition to the use of Arista over the gallbladder fossa to ensure adequate hemostasis. No evidence of a bile leak was detected. The gallbladder was then removed from the abdomen without complications. The umbilical port site was closed with 0-Vicryl. 4-0 Monocryl was used to close skin at all port sites. All incisions were then dressed with Dermabond. Upon completion of the procedure, the patient was taken off anesthesia, transported to the post-anesthesia care unit for recovery, and subsequently discharged home without any acute postoperative complications.

## Discussion

In this case report, we highlight the added layer of complexity that comes with managing SIT patients who present with traditionally more common diagnoses like symptomatic cholelithiasis. SIT is a clinically asymptomatic congenital condition that involves the visceral organs being located directionally opposite of the typical human anatomy. Though multiple theories exist, the etiology of this autosomal-recessive condition remains unknown [3]. Despite the location change, all affected organs function as intended [6]. SIT patients present to the clinic with an added layer of complexity for physicians. When diagnosing and managing these patients, seemingly common abdominal pathologies will present in traditionally different anatomical locations, requiring a more thorough workup. In many cases, SIT has been linked with comorbid conditions such as Kartagener syndrome, polysplenia/asplenia, and Ivemark’s syndrome [3].

In the surgical realm, the management of a SIT patient comes with challenges that primarily revolve around the unfamiliar reversal of the abdominal anatomy. Additionally, special considerations must be made to account for potential differences in the vasculature, especially in procedures involving the kidneys [3]. Though vascular variation does not exist in most cases, it is important that this be taken into account during the preoperative planning process to decrease the risk of bleeding [11]. These added complexities result in a higher difficulty index rating, which makes intraoperative complications more likely [2]. Fortunately, in our case, laparoscopic procedures are commonly used in gallbladder disease pathologies. The laparoscopic approach to a cholecystectomy was first performed in 1987 and remains the gold standard today [1]. The choice to perform a laparoscopic cholecystectomy in SIT patients provides surgeons with the ability to use the same technique but requires significant adaptability to seamlessly maneuver around the patient's reversed anatomy [1]. Though the option to perform an open laparoscopic cholecystectomy is available, there have been very few reported cases as the laparoscopic approach remains heavily preferred [3]. Other less-invasive approaches have emerged, such as the utilization of robotics, a two-surgeon approach, and a "single-incision" approach; however, none have been attempted enough to become the new gold-standard approach [7-9]. Ultimately, the safety of patients and the optimization of their outcomes are vital. Though experimental approaches may emerge, it is important that surgeons select the technique that allows them to perform the procedure confidently without sacrificing patient safety.

The surgical planning for laparoscopic cholecystectomy in SIT patients requires a strong understanding of their anatomy and additional planning from the surgeon to determine the optimal placement of surgical equipment, staff, and ergonomics to maximize positive outcomes. On the day of surgery, our team placed the lead surgeon on the right side of the patient, and to the left were the first assistant and camera driver. Varying configurations have been reported, with the most common setup placing the surgeon and camera driver on the right of the patient and the first assistant on the left [1]. In addition to optimal positioning, it is advised that the surgeon spend additional time orienting themselves to the anatomical structures within the operative space. Though uncommon, the risk of iatrogenic injuries to the common bile duct has been reported in two laparoscopic cholecystectomy cases with comorbid SIT [2]. Other complications, such as bleeding, may occur, and hemostasis should be accomplished utilizing the same techniques as in a standard patient. When performing surgical planning, it is pertinent that the surgeon take these factors into account and determine which surgical approach is the safest option for the patient. With the inverted anatomy, a surgeon's dexterity and coordination are put to the test. Performing the surgery using an open approach or converting to an open approach is always an option in cases where there is severe inflammation, unforeseen obstructions that require better visualization, extensive adhesions, and anatomical variations that were not accounted for [5]. Preoperative ERCPs can be ordered to help prevent conversion to open surgeries, yielding a 63% success rate; however, they are not commonly ordered unless otherwise indicated [10].

Though multiple approaches to treat symptomatic cholelithiasis exist, laparoscopic cholecystectomy is still reported as the safest and most accepted approach when treating SIT patients [11]. By making slight modifications to the placement of trochars and the surgical team, spending more time preoperative planning, and taking the time to orient to the patient's inverted anatomy, surgeons can continue to perform this traditionally safe procedure in SIT patients without significant fear of inducing harm and prolonging the surgical process.

## Conclusions

The diagnosis of symptomatic cholelithiasis in patients with SIT presents surgeons with a rare and challenging opportunity to perform a traditional laparoscopic cholecystectomy with special modifications that accommodate this patient population's anatomical deviation. Our case discusses the unique considerations that should be taken into account for SIT patients presenting with left upper quadrant symptoms probable for gallbladder pathology. If suspected, ultrasound remains the gold-standard confirmatory diagnostic tool. Meanwhile, a laparoscopic cholecystectomy continues to be the preferred method for surgical intervention. No significant modifications need to be made to the preoperative planning process; however, a strong understanding of the patient's uniquely inverted anatomy should be established. The use of radiographs and CT imaging can be ordered to supplement this. During the procedure, the strategic placement of trocars and the positioning of the surgical team are required to optimize patient outcomes. All in all, a surgeon's adaptability is paramount to the success of this routine procedure due to its deviation from standard practice.
